# Barriers and facilitators to adherence to group exercise in institutionalized older people living with dementia: a systematic review

**DOI:** 10.1186/s11556-018-0200-3

**Published:** 2018-11-09

**Authors:** Jitka Vseteckova, Manik Deepak-Gopinath, Erica Borgstrom, Caroline Holland, Jan Draper, Yannis Pappas, Eamonn McKeown, Klara Dadova, Steve Gray

**Affiliations:** 1 School of Wellbeing and Social Care, Faculty of Wellbeing, Education and Language Studies, The Open University, Walton Hall, Milton Keynes, MK7 6AA UK; 2 Institute for Health Research, University of Bedfordshire, Luton, UK; 3 Health Services Research & Management Division in the School of Health Sciences at City, University of London, London, UK; 4 Department of Adapted Physical Activity and Sports Medicine, Faculty of Physical education and Sport, Charles University, Prague, Czech Republic

**Keywords:** Adherence, Barriers, Facilitators, Group exercise, Ageing, Dementia

## Abstract

**Objectives:**

Research suggests targeted exercise is important for people living with dementia, especially those living in residential care. The aim of this review was to collect and synthesize evidence on the known barriers and facilitators to adherence to group exercise of institutionalized older people living with dementia.

**Methods:**

We searched all available electronic databases. Additionally, we searched trial registries (clinicaltrial.gov, and WHO ICTRP) for ongoing studies. We searched for and included papers from January 1990 until September 2017 in any language. We included randomized, non-randomized trials. Studies were not eligible if participants were either healthy older people or people suffering from dementia but not living in an institution. Studies were also excluded if they were not focused on barriers and facilitators to adherence to group exercise.

**Results:**

Using narrative analysis, we identified the following themes for barriers: bio-medical reasons and mental wellbeing and physical ability, relationships dynamics, and socioeconomic reasons. The facilitators were grouped under the following thematic frames: bio-medical benefits and benefits related to physical ability, feelings and emotions and confidence improvements, therapist and group relationships dynamics and activity related reasons.

**Conclusions:**

We conclude that institutionalized older people living with dementia, even those who are physically frail, incontinent and/or have mild dementia can demonstrate certain level of exercise adherence, and therefore can respond positively to exercise programs. Tailored, individually-adjusted and supported physical activity, led by a knowledgeable, engaging and well communicating therapist/facilitator improves the adherence to group exercise interventions of institutionalized older people living with dementia.

**Electronic supplementary material:**

The online version of this article (10.1186/s11556-018-0200-3) contains supplementary material, which is available to authorized users.

## Background

According to the most recent Dementia UK report, approximately 95% of people with dementia in the UK are aged 65 years and over [1]. Currently, majority of people (63.5%) with dementia in the UK live in the community and the remainder in long term care facilities. Given that the prevalence of dementia increases with age, the proportion of people with dementia residing in care homes rises from 26.6% among those aged 65–74 to 60.8% for those aged 90 and over [2]. As the Dementia UK report indicates, severity of dementia is also linked to increasing age. There are therefore serious implications for quality of life (QL) and care of those living with dementia in long term facilities [1].

Dementia is characterized by a range of cognitive impairments such as difficulty in speaking, communicating and understanding, memory loss, and disorientation in space and time. Some people may also present behavioral symptoms that make them more likely to enter residential care [3]. Increasing need for support and care is likely, as people with dementia experience a progressive difficulty in coordinating and carrying out day-to-day activities including self-care. Care home residents, in addition to cognitive impairment, are likely to have co-morbidities that require multiple medications; significant limitations in carrying out day to day activities including self-care; high levels of depression; and nutritional issues [4].

Exercise interventions carried out with people living with dementia in care settings suggest there is potential for exercise to improve physical function [5], mobility [6] and to slow down decline in performance of activities of daily living (ADLs) [5, 7]. Interventions carried out with nursing home residents (including people with mild cognitive impairment) reported improvements in gait speed and muscle strengthening of the lower body [8]. Such improvements might directly enable the person to become mobile and carry out day-to-day activities including self-care independently or with little assistance.

However, the effects of all interventions depend highly on exercise adherence [9]. The challenge of maintaining sustained participation in physical activity is recognized at policy level [10]. High drop-out rates make the benefits of exercise inconsistent. Recent research suggests that adherence to targeted exercise is variable amongst older adults in general [11]. Rolland et al. [5], and Forster et al. [7] have reported substantially variable adherence rates to exercise interventions in long term care settings ranging from 50 to 97%. Eggermont et al. [12] noted mood improvements amongst nursing home residents with dementia who attended at least 80% of the hand movement exercise sessions. Adherence to a multi-exercise program comprising aerobic, strength, flexibility and balance training predicted improved change in Activities of Daily Living (ADL) scores for those nursing home residents with dementia who participated in 60 or more sessions [12]. Frandin et al. [8] further emphasize the need for continuous, individually adjusted and supported physical activity as crucial for the maintenance of physical function in older adults living with dementia.

A more recent study explored predictors of attendance to group exercise amongst older adults living in care settings [13]. Their research confirmed the role of both individual and institutional factors in influencing adherence to group exercise interventions. Depression, social engagement, socio-economic status of residents, and the presence of an activity coordinator were associated with exercise adherence in residential settings in the UK.

However, we know little about the barriers and facilitators to targeted exercise specifically amongst people living with dementia in care settings despite growing support for their inclusion in exercise programs [14]. Same authors emphasize that, as with older adults more generally, those living with dementia value participating in activities that are meaningful to them. Yet cognitive impairment, often coupled with other health conditions and the need for support, suggests that people with dementia living in care settings might find it more difficult to maintain high adherence rates.

Hence, we conducted a systematic review of the existing literature to collect and synthesize the evidence on known barriers and facilitators to adherence of institutionalized older people living with dementia to group exercise including walking groups.

## Material and methods

A systematic literature search was applied. Wider literature was also scoped to identify the most relevant terms in what seems to be a broad spectrum of participants and interventions related to barriers and facilitators to adherence to group exercising in institutionalised older people living with dementia.

Following an agreement on the final scope of the review, a systematic literature review of studies appraising the existing evidence in practice research literature was devised and conducted. Searches were conducted around barriers and facilitators to adherence to group exercising in institutionalised older people living with dementia.

Heterogeneity of outcomes and other PICO criteria were assessed. As the heterogeneity was found to be high, a narrative synthesis approach was used, using thematic analysis for categorizing data. Narrative synthesis is a commonly used method to synthesise data in the context of a systematic review [15, 16], especially as we anticipated appraising mixed methods (qualitative, quantitative and mixed) studies. Thematic analysis provides the means of identifying relevant themes (based on the review question) across large and diverse bodies of research [17]. The PICO (population, intervention, control, and outcomes) framework was used for framing the inclusion and exclusion criteria (see below).

- Participants: Institutionalized older people living with dementia, worldwide
- Intervention: group exercise, both indoor and outdoor, including walking
- Control: not applicable
- Outcomes: Barriers and facilitators to adherence to specific interventions: attendance rates & dropout rates (where available); main focus on barriers and facilitators to adherence; predictors of adherence.

### Types of studies

The searches were not limited to a specific study design. Hence, all types of study designs, qualitative, quantitative, and mixed-methods, were included in the review for as long as they were focusing on evaluating the effectiveness of group exercise activity in improving physical, social and mental wellbeing of people living with dementia and studies mentioning adherence enough to answer our question. Apart from qualitative studies a whole range of quantitative studies were included in our searches such as randomised, cluster-randomised or quasi-randomised controlled trials, cohort studies, before-and-after studies and interrupted time series. Journal articles as well as conference proceedings were included in the search.

### Other criteria

Studies from around the world were included for as long as an abstract and the paper were written/available in English.

Studies not reporting on participation on physical exercise group activities in institutionalised older people living in residential care with dementia were excluded. Studies not reporting on barriers and facilitators to adherence to such exercise were excluded. Due to limited evidence available studies were not excluded if participants suffered from specific forms of dementia. Search terms included AD and other forms of dementia.

### Analysis

We conducted narrative analysis as heterogeneity of findings was found to be high.

### Search for literature

We searched electronically the following databases: MEDLINE(Ovid), The Cochrane Central Register of Controlled Trials (CENTRAL) (Wiley), PsychINFO (Ovid), Educational Resource Information Centre (ERIC) (Ovid), Cumulative Index to Nursing and Allied Health Literature (CINAHL) (Ebsco), Web of Science Core Collection (Thomson Reuters), Trial registries (clinicaltrial.gov, and WHO ICTRP) search for ongoing studies, SCOPUS, Google Schol ar, and Web of science.

We used the strategy and keywords outlined in Additional file 1: Appendix 1. Databases were searched from January 1990 to 30th September 2017. We searched for and included papers in any language. For all included studies, we searched reference lists. We also searched the list of references of other relevant systematic reviews identified whilst running the electronic searches.

### Selection of studies

Titles and abstracts were screened for eligibility. For any references where authors were unsure whether the study met inclusion criteria, a full text of the article was obtained to aid decision-making and ultimately used a third author as an arbiter where uncertainty remained. The full-texts of all articles that appeared eligible for inclusion were retrieved. Study authors were contacted about unclear or missing information.

### Data extraction and management

Two reviewers independently appraised each of the included studies using a structured critical appraisal tool the Critical Appraisal Skills Programme (CASP) tools. Critical appraisal forms for mixed methods were tested, such as Mixed Methods Appraisal Tool Version 2011 (MMAT-V 2011) [18] as CASP tools do not include a mixed methods checklist. Both suggested tools were previously standardized, validated and are widely used for systematic review purposes.

Each tool was tested with two full text papers and authors of this paper agreed the CASP tool was the best to work with as it fitted the purpose of this review and offered a good selection to cover the types of methodologies used in each of the included studies. Any discrepancies were resolved through discussion between the three authors. Through the critical appraisal of the included studies it was found that some studies may have some gaps in relation to methodological quality and reporting findings (adherence rates were not always reported etc.) but may include contextually-rich details that contribute to the overall narrative synthesis and answer our research question. CASP assessment was undertaken to ensure transparency in the process.

### Risk of bias assessment

Two reviewers independently assessed the risk of bias for RCTs using the ‘Risk of Bias’ tool [19]. RCTs were assessed for risk of bias using the following domains: random sequence generation; allocation concealment; blinding (participants, personnel or outcome assessors); completeness of outcome data. Judgements concerning risk of bias for each study were classified using “yes”, “no” or “unclear” indicating high, low or unclear risk of bias respectively. The results of the risk of bias assessment were incorporated into the narratives of the review.

### Assessment of homogeneity / heterogeneity

Homogeneity was assessed in terms of study population, intervention characteristics and reported outcomes. Where we detected substantial clinical, methodological or statistical heterogeneity across included studies, we did not report pooled results but instead used a narrative approach to data synthesis. We attempted to explore possible clinical or methodological reasons for this variation by grouping studies that were similar in terms of populations, intervention features or methodological features.

### Data synthesis

Findings with a high homogeneity index were synthesized narratively. As mentioned above, narrative synthesis is a commonly used method to synthesise data in the context of a systematic review, especially when appraising mixed methods (qualitative, quantitative and mixed) studies.

‘Guidance on the Conduct of Narrative Synthesis in Systematic Reviews’ [17] was used for the purposes of this review. Firstly, a preliminary synthesis was conducted to develop an initial description of the findings of included records and to organise them so that patterns across records could be identified. This was followed by the iterative approach of a thematic analysis, where multiple ideas and conclusions across a body of literature were categorised into themes [20].

Data extracted from articles were entered into a Table 1. (see below) involving very brief descriptive synthesis (for full table see Additional file 1). Articles were of mixed research methods and although some of them were not primarily focused on barriers and facilitators to adherence to group exercise in institutionalized people living with dementia, all these articles have been presenting and or discussing widely on this subject. Therefore, these were included in our review.

**Table 1:** Studies included in the review (Full length table is in Additional file 1)

Author and year	Type of exercise	Length of program	Sample details (N)	Adherence rate	Main Barriers	Key Facilitators
Lazowski et al. 1999 [25]	Strength, balance, flexibility and mobility training	4 months 45-min sessions, thrice a week	Residents in long term care incl. Those with dementia (*N* = 96)	Completed: *N* = 68 (*N* = 71%), from those, attendance to exercise units averaged 86% for the FFLTC and 79% for the ROM classes	• Seated motion exercises not challenging enough • Lack of space • Timing of exercise during other activities	• Self-paced exercise tailored to the level of abilities • Smaller classes of (3–5 people), more volunteers to assist
Rolland et al. 2007 [5]	Walk, strength, balance and flexibility training	12 months 1 h session twice a week	Residents of nursing homes with AD (*N* = 67 experimental group; N = 67 control group) Recruited in total *N* = 134	residents Of the 110 exercisers who completed the study (84%), mean rate of attendance was 33.2 ± 25.5% of the 88 sessions	• Behavior disorders (40%) • Unwillingness to continue (35%) • Acute disease (15%)	• Relationship between the therapist and participants • Quality of care
Galik et al. 2009 [21]	Functional activities and exercise for older people with dementia	Not described	Nursing home residents with dementia Recruited: N = 7	Not reported	• Behavioral issues (anxiety, agitation) • Medication • Communication breakdown • Fear of injury	• Understanding interests and values of residents • Self-paced activities • Availability of staff • Families involved
Resnick et al. 2009 [22]	Self-efficacy based care intervention classes	6 weeks	Residents of nursing homes total N-486 (*N* = 256 experimental treatment group vs. *N* = 231 control group) At 4 months follow up: *N* = 413 At 12 months follow up *N* = 326	After 12 months *N* = 168 in treatment group (66%), *N* = 158 control group (68%)	• Understaffing/low levels of staffing	• Self-efficacy based motivation • Joining exercise for residents
Frandin et al. 2009 [8]	Individually tailored activities	12 weeks 93 min a week Personalised activity programme	Nursing home residents (*N* = 322), 170 intervention group, 152 control, At 3 months follow up: *N* = 266 residents At 6 months follow up: *N* = 241 residents	After 3 months *N* = 143 intervention group (84%) vs. *N* = 123 control (81%) group, drop out: 27 intervention group vs. 29 control group	• Time specific nature of intervention • Inability to continue exercise themselves • Illness and hospital admissions	• Intervention that supports personal skills, self-confidence • Personalized goals • Constant support • Supervised PA
Finnegan et al. 2015 [13]	Group exercise sessions including walking and dancing	12 months twice a week	Nursing home residents incl. People with CI (*N* = 428)	302 subjects out of 428 completed the study (71%), attendance rate for group exercises was 54,2%	• Depression and frailty • Lack of staff • Socio-economic status	• Perception of exercise benefits • Presence of a dedicated staff • Enjoyment and social engagement
Fleiner et al. 2015 [24]	Strength and endurance program	2 weeks 4 - day structuring sessions each 40 min	People with dementia hospitalized in a hospital, targeted (*N* = 130)	Not reported	• Necessity of exercise session organization according to hospital routines	• Flexible exercise schedules that consider mood variations and actual motivation
Olsen et al. 2015 [23]	Balance and strength exercise Small groups Individually adapted Supervised	10 weeks 3 sessions a week 50–60 min each	8 nursing home residents with dementia (N = 12) Completed: *N* = 8	On average 77% attendance rate, ranging from 47% (2 subjects) to 100% (1 subject)	• Functional limitations • Nursing home routines	• Challenging and enjoyable exercise • Voluntary participation • Instructor skills, engagement, relationship to clients
Tobiasson et al. 2015 [26]	Exergames (videogaming with exercise) 3 h 2–3 times a week	12 months 3 h, 2–3 times a week	Residents of dementia special care units (*N* = 22)	Not reported	• Caregivers’ participation limited • Design issues - handling the video game systems	• Enjoyment from playing games and competition • Embedding activity in care giver routine

## Results

Initially we have been looking for articles including only barriers and facilitators to adherence to walking groups in institutionalised people living with dementia. However, we did not find any literature focusing on walking groups, therefore we have widened the search to any exercise group.

Based on our search, we have identified 9 research articles (for details see Table 1.) on different types of group exercise focusing on nursing/residential care homes people with dementia, relating to barriers and facilitators to exercise adherence. Altogether these trials included *N* = 1630 participants recruited and *N* = 1084 who completed including the follow up at maximum length. (For more details see the Table 1. below).

The findings are further structured and presented under the headings of barriers to adherence and facilitators to adherence.

### Barriers to adherence

Known barriers to adherence to group exercise interventions reported in the included studies were grouped in three thematic categories: bio-medical reasons and mental wellbeing and physical ability; relationship dynamics; and socioeconomic reasons, within which they were then listed in alphabetical order.

*Physical health and mental wellbeing related reasons*

- Acute disease [5]
- Anxiety and agitation, depression [13, 21]
- Being cognitively more intact - milder stage of dementia [8]
- Fear of injury [21]
- Frailty including symptoms of muscle weakness [13]
- Increased disability in ADL [5]
- Low levels of previous physical activity and slow walking speed [13]
- Medication [21]

*Relationship dynamics*

- Disagreement within the groups or unwillingness to continue [5]
- Family expectations and communications [21]

*Socioeconomic reasons*

- Low staffing levels [21, 22]
- Socioeconomic status (SES), the lower the more of a barrier [13]

### Facilitators to adherence

Known facilitators to adherence to exercise group interventions reported were grouped in the following themes: bio-medical benefits and benefits related to physical ability; feelings and emotions and confidence improvements; therapist, staff and group relationship dynamics; and activity-related.

*Bio-medical benefits and benefits related to physical ability*

- Physiological benefits, improvements in physical well-being, ‘*pushing the limits*’ [13, 23]
- Skills improvement [8, 13]

*Feelings and emotions and confidence improvements*

- Mastery of exercise [13], empowerment, psychological well-being, self-worth, enjoyment and achievement linked to self-efficacy [13, 23]
- Regaining control [13] and increased independence and improved self-esteem [23]

*Therapist, staff and group relationship dynamics*

- Anticipating challenges, being prepared; giving written instructions where necessary; using assistive devices wherever necessary [21, 22]
- Availability of staff [21]
- Knowing the person’s past; humour and play; short clear verbal cues; repetition; communicating; giving attention [21, 23]
- Motivating nursing assistants as well as the residents to engage with physical activities [22, 23]
- Presence of an activity coordinator or therapist [13] and their competence, trustworthiness and knowledgeability [23]
- Respecting institutions’ routines and the intra-daily variability of the patients’ motivation and behavioural disturbances [8, 24]
- Small numbers (three to five people ideally plus volunteers to assist the therapist/instructor) [25]
- Social interaction, communication and relationships within the group and with the therapist/instructor [5, 23]

*Activity related*

- Allowing space for gaming approach (light competition for example) where appropriate in a socially encouraging environment [26].
- Flexible scheduling and voluntary participation [21, 23, 24]
- Tailoring the activity and its safeness [23–25]; setting realistic individual goals and targeting improving independence [8] moderating appropriate activity ‘*dosage’* whenever appropriate [23, 24]; allowing space for individual uptake of the exercise: ‘*Letting them do their own thing at their own pace’* [21], challenging if necessary: ‘S*omething challenging in otherwise undemanding environment’* [23]

### Adherence rates

Though we have not intend to investigate the adherence-rates per se, studies included in the review reported either data about adherence (percentage of patients who finished the exercise program) or about attendance rate (number of exercise sessions attended, divided by the number of exercise sessions offered). Adherence ranged from 84% (Frandin et al. 2009) to 25.5% (Rolland et al. 2007) with high interindividual variability.

## Discussion

There was variation in the reported factors relating to barriers to adherence to participation to group exercise for older institutionalized people living with dementia. This discussion is structured around the main themes resulting from the narrative analysis: barriers, facilitators, ambiguous findings, and predictors of attendance and limitations.

### Barriers

Mental wellbeing, anxiety, depression (including low morale) and decreased activities of daily living (ADL) have been mentioned several times as barriers for adherence to exercise organized and facilitated for institutionalized older people living with dementia. Recent research suggests that two out of five institutionalized older people living with dementia are depressed [13] and this has a negative effect on adherence to exercise programs [13] and ADL. Low morale as part of depression has also been associated with increased risk of mortality in older people [27]. According to Finnegan et al. [28] social engagement and socio-economic characteristics were significantly associated with participant attendance at exercise groups in the residential homes as well. None of these factors were identified as predictors for adherence to group exercise [13] by this review. The link between depression and adherence has been corroborated by other authors such as Underwood et al. [28] who claim that levels of depression are likely to rise when attendance to exercise group is low, or that a ‘simple’ exercise program, defined as one hour of exercise twice a week, led to a significantly slower decline in ADL and depression scores in institutionalized older people living with dementia [5]. It has also been suggested that the attainment of positive mental health and decreased depression also depends, in considerable part, upon an individual’s self-efficacy – the belief that one can organize and execute the courses of action required to develop and enhance a person’s belief that he or she can act in ways that lead to a desired goal. An intervention suggested that to boost mental health wellbeing for people suffering from depression, sadness and loneliness would be to develop and enhance self-efficacy [29].

Physical limitations such as pain, fear of falling and comorbidities were not identified as common barriers to exercise and adherence to exercise. A reason for this might be that all residents included in the studies had been living with some of these physical limitations for quite some time [13].

Residents’ socioeconomic status (SES) was another barrier identified by our review. SES was a significant predictor of attendance to group exercise [13]. Withall et al. [30] similarly suggests that economically disadvantaged individuals are less likely to engage with exercise interventions [30].

The finding that the more cognitively intact individuals dropped out after the first few exercise sessions, as reported in Frandin [8], is interesting. An explanation for this might be that individuals were more likely to intentionally stop participating because they still had the intellectual capacity to make the decision to do so, while those residents who were severely cognitively impaired were likely to be incapable of making such a resolute decision [8]. This reinforces the importance of continuous physical exercise being adjusted to the functional level and needs of each resident and supported by rehabilitation staff. This approach is crucial for the maintenance of the best possible physical function in these vulnerable elderly persons [8, 21, 23, 25]. Tailoring the group exercise, regardless of the type of exercise, has been identified as a powerful facilitator (discussed below).

### Facilitators

Where residents showed adherence to group exercise, the literature suggests this was due to the physiological benefits (improvements in physical well-being), psychological well-being, feelings of enjoyment and achievement linked to skills improvement, improved self-efficacy and mastery of exercise [8, 13, 23]. Regaining some control and sense of self-worth are also a possible explanation for participants’ attendance [13, 23].

Self-efficacy was reported to be linked with social engagement and support from others (e.g. initiating interaction with other residents and pursuing involvement in the life of the facility) can be related to self-efficacy or the residents’ beliefs in their own ability to complete tasks and achieve goals [13]. This is an important finding as perceived self-efficacy is defined as people’s beliefs about their capabilities to produce designated levels of performance that exercise influence over events that affect their lives. Self-efficacy beliefs determine how people feel, think, motivate themselves and behave. Such beliefs produce these diverse effects through cognitive, motivational, affective and selection processes [31].

Several quantitative studies have already established the positive effects of exercise on biopsychosocial factors, such as self-efficacy in older people, or qualitative aspects of participating in an exercise program among older people with dementia [13, 23].

The exercise program has been reported to improve self-efficacy through several mechanisms. By ‘*being involved*’, ‘*being invested in’* and having ‘*something expected of them*’, the participants gained a sense of empowerment and self-efficacy in their everyday lives [23].

Facilitators can relate to external factors (program, access, instructor etc.) but also to factors that have to do with feeling positive outcomes from participating in it. Exercise revives the body, increases independence, improves mood and self-esteem [8, 13, 23]. Older people participating in Olsen et al.’s study [23] felt their body became more alive and vital, that their energy level increased and they were more content. Exercising had a positive effect on motor function and ADL performance [23]. The participants reported it was easier to rise from a chair, to walk and to climb the stairs after engaging in exercise [23]. Exercise increased the feelings of security and self-esteem and improved self-efficacy [23]. These findings were also suggested by other authors in earlier studies [32]. Also, as noted earlier, improved self-efficacy is also associated with improved depression scores [5, 28]. It is interesting that studies are not dedicated more to such important factors as time and place of the exercise program (organizational aspects).

Another facilitator identified in the literature was motivation. Using motivational techniques has been reported as important not only in terms of motivating the participants but also motivating the staff to join the participants in their activities [22]. However, motivating staff is not without practical challenges, especially as most long-term care institutions are currently understaffed [21]. Having enough staff to engage with participants in their activities is rather rare. Our review identified that wherever staff (physiotherapists included) engage and support vulnerable older people living with dementia the best possible physical function in this particular group is seen [8, 21]. Thus, the availability of staff can work as both a facilitator or a barrier. The importance and benefits of staff participation and engagement have been discussed to a large extent by Galik et al. [21] and Cohen-Mansfield et al. [33].

Another strong facilitator in terms of adherence to group activity is the presence of an activity coordinator/facilitator/therapist [5, 8, 13, 21–23, 25, 26, 33]. Studies report the characteristics that such a person should ideally have so that they positively affect the adherence rates to group exercise. Knowledgeability in terms of exercise, but also of the participants, and an ability to engage and communicate well with the participants were most often cited [5, 13, 21–23, 33]. This places significant importance on relational aspects and dynamics within the group and also between the participants and the therapist/facilitator/staff member. It was also suggested to keep groups with small numbers of participants [25, 33].

Having a positive relationship with other residents and with the physical therapist appears to facilitate exercise participation [23, 33]. The therapist’s ability to adjust and accommodate the exercises to the participants’ needs during the sessions, offering verbal clues and writing down the instructions, if necessary [21] was also important. An appreciation is needed that older people are heterogeneous, and one cannot make the same demands on everybody. The therapist must possess this knowledge, be observant, and be able to make adjustments to the exercise program while instructing [23]. Communication between the therapist and participant also seemed important to the residents [23, 33].

Knowledgeability and good communication from the therapist towards the participants facilitates appropriate tailoring of group exercise and tailoring the activity was shown to be an important facilitator [8, 23, 25, 33]. It was understood by all above cited authors, that moderating appropriate dosage of exercise, however, is something that cannot be prescribed by a manual [23, 25]. It has been pointed out by the above authors, in agreement with the participants and therapists/facilitators, that tailoring the intensity minimizes frustration and boredom, while optimizing the level of challenge [23, 25, 33].

The above discussion has highlighted the barriers and facilitators to adherence in institutionalized older people living with dementia. There was also some ambiguity in the findings with tailoring and ‘*dosage’* of the activity being identified as both a facilitator or a barrier.

### Ambiguous findings

Some residents reported that participating in a group exercise more than twice a week would make them drop out [23], while some other participants reported that it was good to have some challenges in an otherwise ‘*undemanding environment*’ [23].

It is therefore clear that individual abilities and preferences need to be considered while tailoring the best and appropriate level of exercise in order to enhance adherence to this exercise. This is one of the challenges of creating the most ‘adherable’ program for such a heterogeneous group. This, as a limitation, needs to be considered in future empirical research.

Another ambiguous finding was that some participants just want to be left doing whatever they like doing – ‘*letting them do their own thing at their own pace’* [21] and too much coaching would make them drop out. Conversely, other participants require a higher level of stimulation or coaching if they are to adhere regularly to the group exercise [21, 23]. These two opposing positions are not easy to accommodate within one session. Therefore, to attain the highest adherence to the exercise it might be appropriate to split the group according to individual preference. This emphasizes again that, to tailor an exercise program for institutionalized older people living with dementia, it must be done in a personalized, person-centred way that takes into account people’s preferences individual needs in that moment.

### Predictors of adherence

Some authors included in the systematic review attempted to also evaluate predictors of attendance, while others did not. For example, Finnegan [13] suggested predictors of attendance to group exercise included lower depression scores, perceived social support and active involvement in the home and their influence on self-efficacy and home, level socio-demographics and environmental constraints. However, Finnegan [13] also commented that none of the observed variables was actually predictive in relatively small samples in the nursing homes. Therefore, further research is needed.

Benito-Leon et al. [27] suggested low morale as an indirect predictor of adherence because of the association between morale and mortality. By assessing morale, practitioners and researchers might be better positioned to identify patients with poorer prognoses [27] therefore indirectly predicting more likely dropouts.

As indicated earlier, residents’ SES was a significant predictor of attendance to group exercise [13, 30]. Economically disadvantaged individuals were reported to be less likely to engage with exercise interventions [30]. One explanation for this might be that the perception of exercise is a complement to our wellbeing. For generations that had to deal with wars, famine and other adverse circumstances, people and especially those from lower SE backgrounds were more focused on bare survival. Doing exercise for leisure has become a trend in recent years especially for those who do not suffer from poverty.

The findings form this review indicate that tailoring exercise sessions for institutionalized older people living with dementia can only be done by a knowledgeable therapist who can well and effectively communicate with the participants. For this particular group, their individual abilities and preferences need to be considered and accounted for together with leaving the participants with choice and some degree of flexibility. How this can be best achieved is as yet unknown and needs to be considered in future empirical research.

Another finding relating to institutional challenges to provide such tailored groups with such knowledgeable therapists and motivated staff is the fact that most nursing homes in this paper were struggling with staffing levels, as is very common at this level of care globally. Lacking resources mean that is it practically very difficult to form such therapeutic groups, dedicating sufficient numbers of therapists and staff members to ensure smooth running. This of course influences back the motivation of the residents and their adherence. The lacking staffing and financial resources are a barrier that has to be taken into consideration.

### Limitations

Apart from one paper, the main focus for most papers was not directly on barriers and facilitators to adherence to walking group activities. Some of the included papers were also of poorer methodological quality. We have, nevertheless, included these in this systematic review as these were still providing some useful answers to what the known barriers and facilitators to adherence are in institutionalised older people living with dementia. This paper was not methodologically focused, and we were not assessing effectiveness of described interventions, therefore we have included useful information about barriers and facilitators even if the overall methodological quality of some papers was not excellent. Due to this fact and the fact that the amount of literature around interventions including physical activity in this particular group of participants is very limited, we had to also include papers that tangentially mentioned barriers and facilitators to adherence. As mentioned earlier in this section, we were not focusing on effectiveness of the interventions therefore we were not dwelling on adherence rates, as these were not the main focus of this review. However, exercise adherence rates reported in studies included in the review were different in different papers and ranging from 84% (Frandin et al. 2009) to 25.5% (Rolland et al. 2007). Even more variable were attendance rates ranging from low attendance rates (around 40%) to high (around 80%). This can show how complex and multifactorial exercise adherence is.

As mentioned above another limitation are lacking resources and low staffing levels.

Due to the lack of research in this area with this population (institutionalized older people living with dementia), an in-depth exploration as originally intended was not possible. This lack of literature is an interesting finding itself, which shows a gap in the body of knowledge that requires further exploration.

Behavioral disorders were mentioned as a potential barrier by Rolland et al. [5] and Fleiner et al. [24] but not explored in detail and so not discussed above. Since behavioral disorders can manifest in people living with dementia, exploring its links to adherence might be important in future research.

## Conclusions

This systematic review aimed at barriers and facilitators of adherence to group exercises in institutionalized older people living with dementia. We have reported on the nine papers that met the inclusion criteria.

We conclude that institutionalized older people living with dementia, even those who are physically frail, incontinent and/or have mild dementia, can demonstrate certain level of adherence. Therefore, we presume that they can respond positively to a moderately challenging exercise program or programme that is tailored to their needs.

The main barriers were bio-medical reasons and mental wellbeing and physical ability; relationship dynamics; and socioeconomic reasons. The facilitators were bio-medical benefits and benefits related to physical ability; feelings and emotions and confidence improvements; therapist and group relationship dynamics; and activity related reasons.

In addition, tailored, individually-adjusted and supported physical activity, led by a knowledgeable, engaging and well communicating therapist/facilitator are crucial for the adherence to group exercise interventions of institutionalized older people living with dementia.

## Additional file

Additional file 1: **Appendix 1.** Key words in our searches. **Appendix 2.** Flowchart. **Appendix 3.** Flowchart, Full length table of included studies. (DOCX 60 kb)

## Abbreviations

BMI: Body Mass Index
CENTRAL: The Cochrane Central Register of Controlled Trials
CF: Cognitive Function
CI: Cognitive Impairment
CINAHL: Cumulative Index to Nursing and Allied Health Literature
CMOs: Chief Medical Officers
COP: Centre of Pressure
CVD: Cardiovascular Disease
DQOL: Dementia Quality of Life
ERIC: Educational Resource Information Centre
HREC: Human Research Ethics Committee
MANCOVA: Multivariate Analysis of Covariance
MCA: Mental Capacity Act
MMSE: Mini Mental State Examination
PA: Physical Activity
PICO: Population, intervention, control, and outcomes
QoL: Quality of Life
QOL-AD: Quality of Life in Alzheimer’s disease
SES: Socioeconomic status
WHO ICTRP: World Health Organisation International Clinical Trial Registry Platform
