# Supplementary material for: Barriers and facilitators to adherence to group exercise in institutionalized older people living with dementia: a systematic review
Source: Eur Rev Aging Phys Act. 2018 Nov 9;15:11. doi: 10.1186/s11556-018-0200-3 (PMC6225693; doi:10.1186/s11556-018-0200-3)
Supplement: Supplementary file 1 — Appendix 1. Key words in our searches. Appendix 2. Flowchart. Appendix 3. Flowchart, Full length table of included studies. (DOCX 60 kb) [file 11556_2018_200_MOESM1_ESM.docx]

**Appendix 1. Key words in our searches**

Preliminary search version 1 (February, 2017)

(aged OR elder* OR "older adult" OR "older person" OR "old age") AND (dementia OR "Alzheimer disease" OR “cognitive impairment”) AND ("nursing home" OR "care home" OR “care setting” OR resident*) AND (walk* OR exercise OR “physical activity”) AND (group OR prog* OR initiative OR trial OR scheme OR club OR intervention OR therapy) AND (well* OR benefit* OR welfare OR "quality of life" OR QoL)

Preliminary search version 2 (March/May 2017)

(aged OR elder* OR “older adult” OR “older person” OR “older people” OR “old age”) AND (dementia OR “Alzheimer disease”) AND (“nursing home” OR “care home” OR “care setting” OR resident*) AND (exercise OR “physical activity” OR “aerobic activity”) AND (walk* OR “walking exercise” OR “walking programme” OR “walking program” OR “walking group” OR “walking club”) AND (adherence OR attendance OR “drop out” OR barrier* OR facilitator* OR participation OR experience OR effect* OR well* OR benefit* OR welfare OR “quality of life” OR QoL)

**Appendix 2: Flowchart**

Database Search

N = 1658 papers/citations identified from electronic literature search and screened

Papers excluded following title screening, removal of duplicates and abstracts screening (N = 15)

Grey literature search didn’t yield any potential articles for inclusion

Potentially relevant articles retrieved

N=15

Review of full text papers (n=15)

6 papers excluded:

- Not a research study (3)
- Study protocol (2)
- Intervention did not include institutionalised people living with dementia (1)

9 studies included in the review and assessed by critical appraisal

Reasons for further exclusion:

- (N=0)

**9 studies finally included in the review**

**Appendix 3 Full length table of included studies**

| **Author(s) and year** | **Study design** | **Aim of the study** | **Type of exercise intervention** | **Sample details** | **Main Barriers** | **Key Facilitators** |
| --- | --- | --- | --- | --- | --- | --- |
| 1. Galik et al 2009 | Qualitative study with focus groups | To explore facilitators and barriers to engaging cognitively impaired residents in functional activities and exercise | Functional activities and exercise for older people with dementia | Purposive sample of 7 geriatric nursing assistants caring for nursing home residents with moderate to severe dementia | - Behavioural issues such as anxiety and agitation - Use of medication can inhibit participation; - Communication breakdown amongst staff - Fear of injury for both resident and carer - Focus primarily on behavioural control strategies to minimise agitation - Encouragement of sedentary activities to prevent outbursts | - Understanding what motivates the person taking into consideration their past and present interests and values - Encouraging self-paced carrying out of activities by residents - Interaction between staff and residents not subject to time limitations - Working with residents’ families and wider care teams - Availability of staff |
| 1. Resnick et al 2009 | Randomized, controlled, repeated measure design | To assess the effectiveness of a care intervention (based on restorative care philosophy) on function, strength, contractures and quality of life of nursing home residents | 6 week in service self-efficacy based care intervention classes for nursing assistants of nursing homes with written tests conducted after 6 weeks, 4 and 12 months | Nursing assistants and residents of nursing homes (including those with dementia) | Understaffing/low levels of staffing | - Self-efficacy based motivational techniques to encourage resident participation in self-care and exercise activities - Motivating the staff to join the residents in the exercise |

| **Author(s) and year** | **Study design** | **Aim of the study** | **Type of intervention** | **Sample details** | **Main Barriers** | **Key Facilitators** |
| --- | --- | --- | --- | --- | --- | --- |
| 1. Frandin et al 2009; 2015 | Randomized, controlled trial | To assess the impact of an individually tailored intervention on physical performance, balance, activities of daily living, participation in physical activity, self-efficacy, wellbeing and cognitive function in nursing home residents | Individually tailored activities carried out with individuals or groups for 93 minutes per week over 12 weeks; intervention led by a team of physiotherapist and occupational therapist at each site | Nursing home residents including: those with mild cognitive impairment; those who could walk with or without aids; and, those who could rise from chair independently | - Time specific nature of intervention which may affect adherence once intervention is over - Inability of residents to continue physical training themselves once intervention over - Illness and hospital admissions | - Intervention that supports personal skills, self-confidence and personal goals and values of residents - Setting goals for residents that are personalised, as realistic as possible and adjusted to individual and cognitive capacities - Constant support and encouragement from and access to rehabilitation staff - Supervised physical activity |
| 1. Finnegan et al 2015 | Randomized, controlled trial | To identify predictors of attendance at exercise groups in residential and nursing home facilities | Group exercise sessions including walking and dancing conducted twice weekly for 12 months | Residential and nursing home residents including people with moderate cognitive impairment | - Depression and frailty amongst residents - Lack of activity coordinator and staff resources to bring participants to exercise groups - Socio-economic status associated with participant attendance in residential care homes - Care home culture in promoting activities | - Benefits of exercising as perceived by residents - Presence of a dedicated activity coordinator (residential care homes) - Enjoyment and social engagement indirectly promoted by exercise activity |

| **Author(s) and year** | **Study design** | **Aim of the study** | **Type of intervention** | **Sample details** | **Main Barriers** | **Key Facilitators** |
| --- | --- | --- | --- | --- | --- | --- |
| 1. Fleiner et al 2015 | Study protocol for a randomized, controlled trial | To identify the effect of physical exercise on behavioural and psychological disturbances in people with dementia | 2 - week exercise programme comprising of 4 - day structuring exercise sessions (strength and endurance) of 40 minutes each with group sizes of 3 participants per instructor. | People with dementia hospitalised for behavioural and psychological disturbances in special dementia units within a psychiatry hospital | Organisation of exercise sessions needs to respect hospital and nursing care routines. | - Organisation of exercise sessions to respect hospital and nursing care routines - Flexible exercise schedules that consider mood variations and motivation of participants |
| 1. Olsen et al 2015 | Qualitative study with participatory design | To explore the experiences of participating in an exercise programme amongst nursing home residents with dementia | 10 - week group exercise programme (small groups) individually adapted comprising of balance and strength exercise conducted in three sessions per week of 50-60 minutes each; supervised by a physiotherapist | 8 nursing home residents with dementia | - Functional limitations - Nursing home routines | - Exercise that is challenging and enjoyable - Voluntary participation at each session - Being able to complete an exercise session a motivating factor - Instructor skills, competence and engagement important for encouraging and adapting exercise as required - Trusting and respectful relationships with instructor - Staff attitudes about capabilities of people with dementia to exercise - Social aspects of group exercise |

| **Author(s) and year** | **Study design** | **Aim of the study** | **Type of exercise intervention** | **Sample details** | **Main Barriers** | **Key Facilitators** |
| --- | --- | --- | --- | --- | --- | --- |
| 1. Lazowski et al 1999 | Randomized, outcome evaluation | To evaluate whether targeted group exercise is more beneficial than a range of seated motion exercises | 45 minute sessions with 4-10 participants per group; Exercise included strength, balance, flexibility and mobility training conducted thrice a week for 4 months; recreation staff, aides and volunteers trained to assist with exercise class | Residents in long term care including: those with dementia; those who required aids and assistance for mobility; and, those who could move around independently | - Seated motion exercises not challenging enough - Delivery of exercise can be challenging with lack of sufficient space in long term settings for exercise activities and if timing of exercise clashes with other activities within the setting - Attitudes towards exercise programmes | - Importance of tailoring the exercise class to different levels of resident abilities - Smaller classes of three to five people and more volunteers to assist the instructor/facilitator - Self-paced and progressive exercise programme |
| 1. Rolland et al 2007 | Randomized, controlled trial | To improve the ability to perform activities of daily living (ADLs) amongst people with Alzheimer’s (AD). | Group exercises with 2-7 participants per group; Group exercise comprising 1 hour twice weekly of walk, strength, balance and flexibility training over 12 months; exercise sessions led by an occupational therapist | Residents of nursing homes with mild to severe Alzheimer’s and who could walk without assistance distances up to 6 meters | - Behaviour disorders (40%) - Disagreement or unwillingness to continue (35%) - Acute disease (15%) - Increased disability in ADLs (5%) - Other several reasons not stated (5%) | - Interaction and relationship between the therapist and the participants - Quality of care in the nursing home as a contextual factor in supporting adherence |

| **Author(s) and year** | **Study design** | **Aim of the study** | **Type of intervention** | **Sample details** | **Main Barriers** | **Key Facilitators** |
| --- | --- | --- | --- | --- | --- | --- |
| 1. Tobiasson et al 2015 | Qualitative study with a participatory design | To introduce exergames in dementia special care units with a view to improving resident wellbeing | Three hours twice a week of exergames (videogaming with exercise) for 4 months at one site and three hours twice or thrice a week for 12 months at other two sites; Care staff trained to facilitate exergames | 22 residents of dementia special care units including those who could be mobile with and without aid s/assistance, those with impaired ability to speak | - Caregivers’ participation limited if activity not a part of caregivers’ routine work - Design issues related to handling and using the video game systems - Organisation attitudes towards activities for people with dementia | - Enjoyment derived from playing games in a socially encouraging environment - Notion of games, competition and challenge meaningful for continued participation - Involvement of entire organisation in the aim of motivating residents to learn, manage and enjoy - Organisational buy in into exergame activity - Embedding activity within care giver daily routines |
